# Extrastriate visual cortex reorganizes despite sequential bilateral occipital stroke: implications for vision recovery

**DOI:** 10.3389/fnhum.2015.00224

**Published:** 2015-04-28

**Authors:** Amy Brodtmann, Aina Puce, David Darby, Geoffrey Donnan

**Affiliations:** ^1^Behavioural Neuroscience, Florey Institute for Neuroscience and Mental Health, University of MelbourneMelbourne, VIC, Australia; ^2^Department of Psychological and Brain Sciences, Indiana UniversityBloomington, IN, USA

**Keywords:** visual cortex, stroke, reorganization, plasticity, hemianopia, fMRI

## Abstract

The extent of visual cortex reorganization following injury remains controversial. We report serial functional magnetic resonance imaging (fMRI) data from a patient with sequential posterior circulation strokes occurring 3 weeks apart, compared with data from an age-matched healthy control subject. At 8 days following a left occipital stroke, contralesional visual cortical activation was within expected striate and extrastriate sites, comparable to that seen in controls. Despite a further infarct in the right (previously unaffected hemisphere), there was evolution of visual cortical reorganization progressed. In this patient, there was evidence of utilization of peri-infarct sites (right-sided) *and* recruitment of new activation sites in extrastriate cortices, including in the lateral middle and inferior temporal lobes. The changes over time corresponded topographically with the patient's lesion site and its connections. Reorganization of the surviving visual cortex was demonstrated 8 days after the first stroke. Ongoing reorganization in extant cortex was demonstrated at the 6 month scan. We present a summary of mechanisms of recovery following stroke relevant to the visual system. We conclude that mature primary visual cortex displays considerable plasticity and capacity to reorganize, associated with evolution of visual field deficits. We discuss these findings and their implications for therapy within the context of current concepts in visual compensatory and restorative therapies.

## Introduction

Serial functional magnetic resonance imaging (fMRI) has been used to examine cortical remodeling following injury and track functional recovery, particularly after strokes involving the motor and sensory systems (Calautti and Baron, 2003). Activation patterns are often compared to those of healthy age-matched control participants, and assume that pre-stroke activation in the patient population would have been comparable to those of controls. There are few case reports in the fMRI literature where individuals were studied prior to their deficits (Thulborn et al., 1999), and sequential strokes have rarely been studied. In addition, researchers studying the visual system have usually recruited patients months to years following their injury (Goebel et al., 2001; Nelles et al., 2002; Schoenfeld et al., 2002). Here we present qualitative fMRI findings from a 49 year old man, who participated in a prospective study of visual reorganization after stroke. He suffered sequential occipital strokes 3 weeks apart affecting first the left, then the right visual cortices. We contrast his first scan at 8 days following his first stroke with that done on review at 6 months, and with those of a healthy control subject selected from a group of people tested for associated projects. We then discuss mechanisms of recovery in the visual cortex following stroke, and present a brief summary of both compensatory and restorative therapies and their implications for rehabilitation.

## Background

### Ethics

Informed written consent was obtained. The study was approved by the Austin Health Human Research Ethics Committee, which abides by the Declaration of Helsinki.

### Participants

Single case data were taken from a prospective clinical study on the fMRI study of visual recovery after stroke. Patients received standard stroke management, including admission imaging with CT or MRI scans. Patients were studied within 10 days post-stroke (Session 1) and at 6 months (Session 2) with fMRI and visual perimetry. Controls had identical testing in two sessions 6 months apart.

### Inclusion criteria

Stroke patients were recruited if they had a confirmed first-ever acute ischemic infarct affecting visual cortex with a corresponding perimetric defect (*n* = 10, one presented here). Other study criteria have been described in detail (Brodtmann et al., 2003, 2007, 2009a,b). Control participants were included if they were free of neurological disease, and aged in the following decade cohorts: 30–39, 60–69, 70–79, and 80–89 years; total *n* = 24, 12 women. This formed the basis of a normal cohort for comparison, from whom we have selected one age- and sex-matched subject for this case report (Brodtmann et al., 2003, 2009a).

### Experimental design

#### Magnetic resonance imaging

#### Activation task

A block-design was used with alternating central presentations of grayscale unfamiliar faces (FACE), scrambled faces (SCRF), and a 50% gray field with a central black fixation cross (GRAY). Stimulus conditions (A = FACE, B = SCRF, C = GRAY) alternated in a repeated ACBC… sequence—see Figure S1 for schematic. Each single stimulus was presented for 0.5 s with no gap between successive stimuli. The duration of each stimulus block was 20 s, therefore 40 FACE or SCRF stimuli were presented in each block. There were three stimulus cycles (i.e., ABCB-) within an imaging run (3 cycles × 20 s block × 4 blocks/cycle = 240 s). The duration of the imaging run was 254 s as it included an initial 8 s non-stimulation (50% gray—rest task) period allowing for steady-state transverse magnetization and a 6 s non-stimulation period (black screen) at the end of the run to allow the hemodynamic response to return to baseline. After a rest of approximately 2–4 min the imaging run was repeated. We have previously demonstrated robust striate and extrastriate activation in the cortices of participants aged 30–90 years using this paradigm (Brodtmann et al., 2003, 2009b).

The subject was placed in the scanner in a supine position with a mirror attached to the head coil centered above his eyes. Through the mirror he was able to see the stimuli projected onto a screen placed at his feet and positioned prior to scanning so that he was able to see the entire image margins. Video images (PAL format size = 768 × 576 resolution) were projected via a mirror through the control room window onto the back of a screen. Once the subject's head was positioned inside the scanner the visual angle of display was approximately 15° in diameter.

##### Functional imaging

All images were acquired on a 3 Tesla GE Horizon LX MRI scanner in a single scanning session (GE Systems, Milwaukee, Wisconsin, USA): 14 coronal T1-weighted slices beginning at primary visual cortex and covering the posterior half of the brain, extending anteriorly through to the thalami. Coronal section was selected as it covered all the visual areas of interest with the least number of slices (compared to axial acquisition, for example). The convoluted nature of the calcarine sulcus means that it is poorly visualized in axial and sagittal slices. Activation on both medial and lateral sides of the fusiform gyrus could also be seen with coronal slices. Fewer slices (i.e., partial brain acquisition) also increased the number of brain volumes per task available for analysis. These images were used as a template for a real-time gradient echo echo-planar imaging sequence (EPI: TR = 2000, TE = 40, FOV = 24 cm, flip angle = 40°, matrix = 128 × 128, in-plane voxel size = 1.85 × 1.85 mm, slice thickness = 4 mm, gap = 1 mm). Two imaging runs were obtained, yielding a maximum number of 60 volumes per condition. A face localizer pre-scan was performed prior to the fMRI scanning run.

#### Behavioral data collection

A target detection task was introduced in order to monitor maintenance of attention on the visual stimulus display. Subjects were asked to detect a transiently appearing centrally presented white cross (target), and to raise their left thumb on target detection. Targets were presented for 0.5 s each time. They were randomly interspersed throughout the paradigm, were equally distributed in each condition, and acted as an objective means of monitoring attention during functional scanning. A total of 12 targets were presented during the course of each imaging run.

Subjects were observed throughout each run for target detection accuracy. Black crosses were not present during the face or scrambled face image presentation displayed without white cross targets. Subjects were requested to keep their eyes directed centrally and remain vigilant for the appearance of the targets. Eye movements were not otherwise monitored during the scanning sessions. The number of hits, misses and falsely identified targets was noted at the end of each imaging run. Student's *t*-test was used to evaluate difference between error rates.

#### Structural imaging

Coronal T1 and magnetic resonance angiography (MRA) sequences and axial 3D SPGR images were also acquired. All structural scans were independently reported by a radiologist blinded to the subject's details so that subtle structural pathology would not be overlooked (e.g., prior silent infarcts, new infarction).

### fMRI data analysis

Our choice of partial brain acquisition for the functional images limited the analysis tools available. MEDx 3.3 (Sensor Systems Inc., Sterling, VA, USA) was used for the majority of the analysis. Motion detection and correction was performed using the AIR algorithm as previously described (Brodtmann et al., 2003, 2007, 2009a,b). Within-subject *t*-test maps were obtained for each comparison of interest i.e., FACE vs. GRAY, and SCRF vs. GRAY. *T*-test maps were thresholded using an uncorrected *p* < 0.001, based on a prior false-positive analysis (Brodtmann et al., 2003). These thresholded maps were overlaid on MRA images to exclude activation arising from large intracerebral blood vessels and onto the matching extracted T1-coronal slices (Smith, 2002). All subsequent analysis was performed using the functional data overlaid onto the extracted T1-images as an index of individual anatomy. We did not normalize or spatially smooth the fMRI data. Data remained in the subject's native anatomical space to allow for differing infarct sites, variable age-related atrophy, possible acute edema and chronic involution.

For each subject, left and right striate and ventral extrastriate cortices were identified a priori, using T1-weighted anatomical coronal sections as a reference and comparing to coronal sections in a standard published text (Duvernoy et al., 1999). Anatomical ambiguities were also clarified by scrutinizing the 3D SPGR high-resolution T1 axial images in the orthogonal viewer in MED × 3.3. Regions of interest (ROIs) were identified, combined into groupings of left and right ventral extrastriate, and left and right striate cortical sites. Each extrastriate region included the posterior two-thirds of the fusiform gyrus, ventromedial parts of the inferior temporal gyrus, inferior lingual, and inferior occipital gyri; the “striate” region included cuneus, gyrus descendens, and the parts of the superior lingual and lingual gyri and precuneus adjacent to the calcarine sulcus. The striate areas were defined according to a standard anatomical reference (Duvernoy et al., 1999).

### Visual field testing

Visual fields were assessed using Medmont M600 automated perimetry including fixation loss errors (Medmont, Camberwell, Australia). This computerized program gives a number of options for assessment. The neurological visual field option was used as this paradigm has been designed to identify a variety of visual field anomalies associated with neurological (as opposed to ophthalmological) disease, including homonymous and bitemporal deficits, scotomas, and cataracts. For the stroke subjects, fields were graded as normal, partial hemianopia, or complete hemianopia. Comparisons of visual fields between sessions were descriptive, graded as minimal, partial, and complete recovery. This is a full field test. Fixation errors were compared between groups, but for the purposes of this case report only individual fixation errors are reported for perimetry.

### Case report

**Figure 1:**
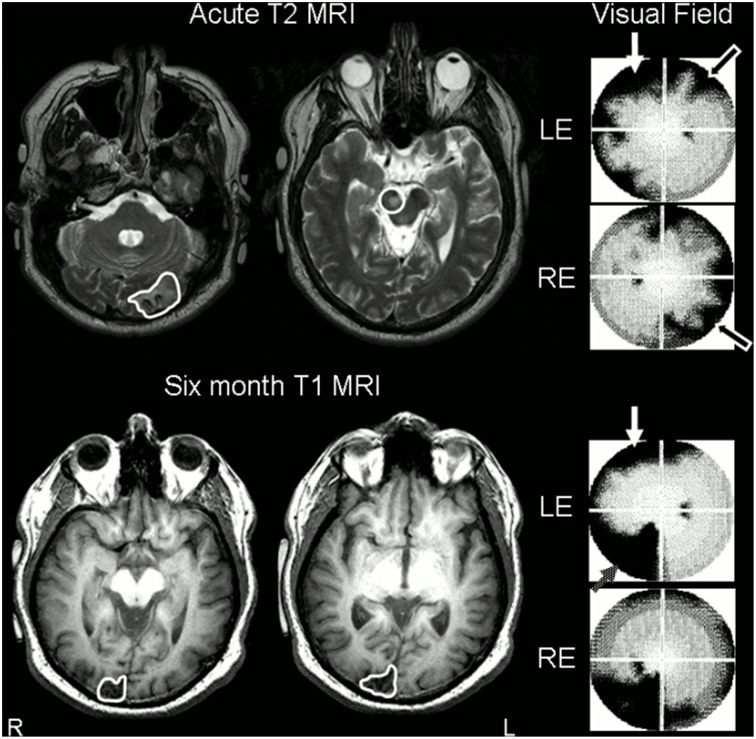
**Stroke sites and corresponding visual field deficits on perimetry. Top Row:** day 8 T2-weighted MRI scan, axial slices (radiological convention, so images are flipped) through occipital cortex showing left acute occipital lesion and demonstrating midbrain lesion (traced or circled in white). Despite the fact that his midbrain lesion was right-sided, the patient had persistent left ptosis, with left lid artifact present on perimetry (white arrow). T2 images are shown as subacute ischemic lesions are difficult to see on T1 images. His right partial homonymous visual deficit can be seen on his perimetric visual field maps (black arrows). Note that perimetric tests were 90° visual field tests. **Bottom row:** repeat MRI at 6 months, axial T1-weighted slices through occipital lobes, revealed interval infarction in the right occipital pole (traced in white). Lid artifact still present from ptosis (white arrow), but perimetry revealed almost complete resolution of right homonymous deficit despite interval development of partial left inferior quadrantanopia (gray speckled arrow). LE, left eye; RE, right eye.

A 49-year-old recreational intravenous drug user with known insulin-dependent diabetes mellitus presented with a 48 h history of visual disturbance, confusion, and reduced hearing following a 2 week period of heavier than usual heroin use. Admission renal function was impaired, presumed secondary to dehydration: creatinine 0.17 mmol/l (normal range 0.03–0.11 mmol/l), urea 17.8 mmol/l (2.8–7.7 mmol/l), glucose 16.1 mmol/l (3.3–8.0 mmol/l); BP was 190/90, heart rate 92. He was disoriented and had trouble hearing the examiner. Left ptosis and a right congruous homonymous visual field deficit with macular sparing were noted. Admission MRI scanning confirmed the presence of a left inferior occipital infarct and right-midline midbrain infarct (see Figure 1).

Blood glucose and renal function normalized rapidly: Day 2 glucose 5.1 mmol/l, creatinine 0.075 mmol/l, creatinine clearance 2.91 ml/s (1.50–2.50 ml/s). Within 2 days his hearing and orientation returned to normal, and his visual deficits rapidly evolved. On day 5, automated perimetry was performed, and revealed incomplete resolution of the right homonymous visual deficit, restricted to the superior quadrant for the left eye and peripheral loss crossing the horizontal meridian in the right eye (Figure 1). Perimetry also demonstrated a patchy symmetrical reduction in peripheral vision in the unaffected hemifield. These deficits have been described previously in the acute post-stroke period, and are believed to be due to associated hypometabolism or diaschisis of the contralesional hemisphere, as they recover over the ensuing weeks after stroke (Bosley et al., 1987). Left ptosis caused significant left lid artifact.

Transcranial and carotid Doppler studies were normal. Contrast transesophageal echocardiography was positive, consistent with a trivial patent foramen ovale, but no significant right to left shunt. Endocarditis was excluded. Stroke etiology was presumed to be thromboembolic secondary to a contaminated intravenous injection. He was discharged home with residual left ptosis.

Two weeks later he noted sudden onset of difficulty reading and reduced vision in the left visual field, not associated with a resumption of drug use. He did not seek medical attention. Repeat MR scanning at 6 months as part of our fMRI research study confirmed the presence of a further right-sided stroke, affecting the superior occipital gyrus and cuneus (Figure 1). After review, the mechanism of this further stroke was thought to be due to non-compliance with stroke prophylaxis with known patent foramen ovale.

### Results

#### Control subject

We chose one age- and gender-matched control (39 year old man) as an exemplar for qualitative comparison for this report. On both occasions, visual field testing was normal, with no fixation errors made on perimetric testing.

#### Patient results

#### Session 1

**Figure 2:**
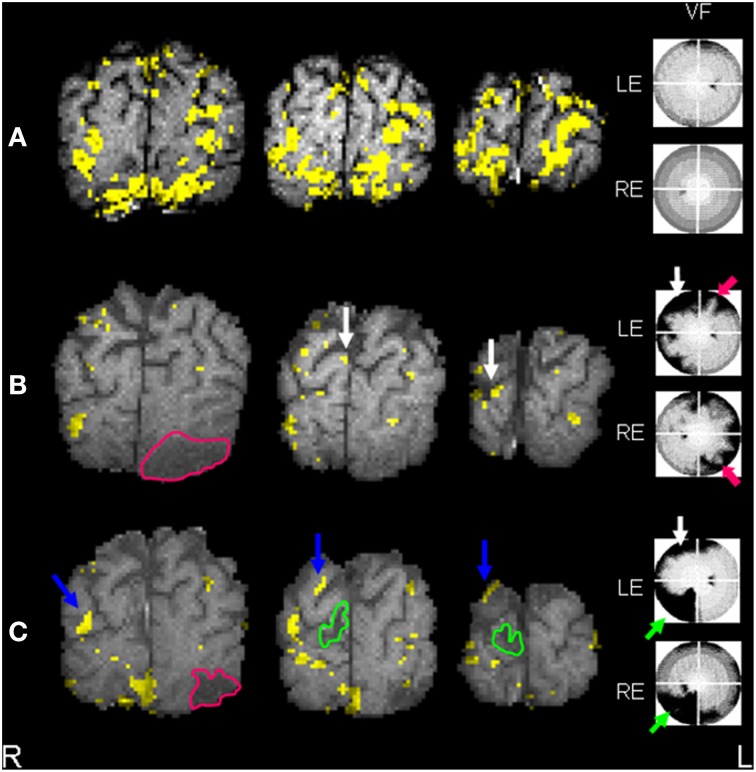
**(A)** Control subject *t*-test SCRF maps (individual exemplar) overlaid onto corresponding T1-weighted images, 3 coronal slices through occipital pole and calcarine cortices, demonstrating symmetrical activation in striate (calcarine) and extrastriate regions. Normal visual field in seen on perimetry, right of figure. **(B)** Patient *t*-test SCRF maps overlaid onto corresponding T1-weighted images, 3 coronal slices through occipital pole and calcarine cortices (radiological convention), demonstrating reduced extrastriate activation and bilaterally absent calcarine activation, common acutely secondary to diaschisis. Stroke site traced in pink. Corresponding visual deficit again shown, highlighted with pink arrow. Note the white arrows highlighting sites of activation in the middle occipital lobe. **(C)** Patient *t*-test SCRF maps overlaid onto corresponding T1-weighted images, 3 coronal slices through occipital pole and calcarine cortices (radiological convention), demonstrating bilateral return of striate (calcarine) activation, despite the presence of a further stroke, stroke site traced in green. Corresponding visual deficit shown, green arrow. Note that the new stroke site has affected an area of activation demonstrated in the initial fMRI, but visual cortical reorganization has continued despite the recurrent lesion. Blue arrows point out regions of activation not present on initial fMRI.

Visual field testing on Day 5 revealed a resolving partial right homonymous hemianopia. One fixation loss was detected for the left eye and four for the right eye with no false positive responses (graded as excellent fixation by Medmont automation). Day 8 fMRI revealed reduced left-sided ipsilesional ventral extrastriate activation (fusiform and lingual gyri) compared to controls, with no evidence of intra-infarct activation (Figure 2). Peri-infarct activation foci were seen medially and laterally. Reduced calcarine activation was seen bilaterally, common in the acute post-stroke period presumed secondary to diaschisis (Brodtmann et al., 2007). Evidence of right superior occipital activation can be seen.

#### Session 2

Visual field testing at 6 months revealed an incomplete resolution of the right homonymous hemianopia and a new left inferior quadrantanopia; one fixation loss and one false positive response were made for each eye. fMRI revealed that fusiform activation was unchanged. On the left (first stroke side) new left superior parietal and left middle temporal gyral activation was noted, with small nodes of peri-infarct activation also apparent. On the right, occipital activation was shifted superiorly and laterally compared to Session I (Figure 2).

## Discussion

### Case report findings

This case report is unique in documenting fMRI evidence of visual system plasticity between sequential occipital infarcts. Initial fMRI evaluation following his first stroke showed abnormal visual cortex activation ipsilesionally and normal patterns contralesionally. 6 months after the initial stroke, left visual cortex reorganization changes were seen along with peri-infarct activation. This report complements one of the few other studies which also document pre-stroke fMRI activation patterns, but which involved a language evaluation rather than visual system paradigm (Thulborn et al., 1999). Whilst there is minimal evidence in the functional MRI literature of patients with recurrent or sequential stroke, we know that functional status declines after ischemic stroke. Recurrent stroke is associated with worse cognition and function (Dhamoon et al., 2012). From this we infer that compensatory remodeling may be limited by recurrent events. Yet the visual system appears capable of functional recovery following repeated infarction (Gray et al., 1989; Goebel et al., 2001; Nelles et al., 2002; Schoenfeld et al., 2002).

There are two main findings. First, contralesional activation was within expected sites and comparable to that seen in controls prior to the second stroke. This finding is important as the major underlying assumption of lesion-based functional neuroimaging studies is that the patient under study *would have had* a pattern of cortical activation prior to injury within the spectrum of normal variation. This patient's data demonstrated the expected pattern of activation in the *right* hemisphere following the initial left occipital stroke.

Second, visual cortical reorganization progresses despite sequential infarcts. In this patient, there was evidence of utilization of peri-infarct sites (right-sided) *and* recruitment of new activation sites, such as the left lateral middle and inferior temporal nodes. The changes over time corresponded topographically with the patient's lesion site and its connections.

### Study limitations

Case reports are always limited by individual variation. In addition, our choice of partial-brain coronal acquisition and inability to register these images into standard space means that only a modest interpretation of the fMRI activation changes is possible. Whilst the patient's visual fields were closely studied, no detailed face perception tasks were performed. Attention was monitored during the activation task, but eye movement monitoring was not performed, meaning that eye movement differences may have contributed to the fMRI changes described.

### Mechanisms of recovery following ischemic stroke

There are two major phases to recovery of function following stroke. The acute phase incorporates re-perfusion with salvage of extant neurons in ischaemic, but not infarcted, zones, and resolution of edema. In this patient, penumbral salvage was not possible as re-perfusion therapies such as thrombolysis or clot retrieval, which need to be given within hours of stroke, were not feasible due to his late presentation. A later or chronic phase includes re-modeling or re-organization of surviving cortex to resume or approximate impaired function. Recovery predominantly occurs in the first few weeks, plateauing at 3 months, but many patients report on-going improvements over years.

Recovery over the first few days is usually due to resolution of edema which is believed to disrupt axonal pathways and hence corticocortical connectivity, thereby uncovering the extent of the deficit (Calautti and Baron, 2003). Reperfusion of the ischemic penumbra appears important, but it is not yet known to what extent this affects prognosis (Astrup et al., 1981; Donnan and Davis, 2002). Some authors posit that the perfusion deficit has more impact on sub-acute post-stroke function than the infarct core (Shirani et al., 2009; Khurshid et al., 2012). In addition, recovery of damaged or ischemic neural networks within the infarct may also contribute, particularly in areas of misery perfusion or chronic ischemia, but these latter two phenomena remain unproven in human studies (Cao et al., 1999; Thulborn et al., 1999). Cellular level changes, such as cortical reorganization via synaptic sprouting, and the formation of new cortical connections may also contribute to recovery (Darian-Smith and Gilbert, 1994).

Patterns of activation after stroke have been reported, characterized by diaschisis, compensation and restitution of function. For example, stroke patients with left motor cortical infarction demonstrate bilateral reductions in motor network activation acutely, presumed secondary to diaschisis. With early recovery, they display increased right motor and supplementary motor activation, which are felt to be compensatory and may relate to more “effortful” tasks. Only with good recovery is there restitution of activation to peri-infarct regions, or even to primary motor cortex in patients with subcortical lesions.

In prior studies examining patients with visual cortical stroke, we have demonstrated similar findings in the visual cortex, with a return of activation to peri-infarct regions with visual recovery. In this patient's first scan, we demonstrated a generalized reduction in right and left hemispheric activation, and have shown this in Figure 2. Whilst reduced, there was evidence of a normal pattern of activation in the right visual cortex. The second right-sided stroke affected the superior occipital gyrus and cuneus, causing a new left inferior quadrantanopia. The second stroke affected some regions that had shown activation in the initial scan. Right visual cortical activation patterns were hence altered in the second scan, exhibiting regions of new or increased activation in the right visual network.

### Relevance to presented case

The information presented in this case report demonstrates evidence of each of these post-stroke phenomena. There was evidence of diaschisis on initial perimetry, associated with a symmetrical constriction in the unaffected hemifield—see Figure 1. Diaschisis may also have contributed to the overall reduction in bihemispheric activation seen on early scanning (Bosley et al., 1987; Brodtmann et al., 2007)—see Figure 2. We interpret the fMRI activation changes as evidence of cortical remodeling, which may be compensatory or associated with a restitution of function. At 6 months, there was almost complete recovery of his right hemianopia caused by his left occipital stroke, with associated return of peri-calcarine (peri-striate) and new extrastriate activation in the left hemisphere. In the right hemisphere, the site of the second stroke, there was increased activation in extrastriate regions, especially within the dorsal extrastriate visual cortex. It is not possible to comment on the recovery of his visual deficit, as perimetry was not performed at the time of the second stroke. We have previously reported utilization of dorsal extrastriate cortices in post-stroke recovery (Brodtmann et al., 2007, 2009b). Dorsal regions are association or “secondary” cortical regions, and demonstrate unique characteristics within the visual network. They are less reliant on striate regulation, display greater inherent levels of experience dependent plasticity, and have faster, more abundant extra-geniculostriate connections (Brodtmann et al., 2009b).

### Mechanisms of recovery following stroke affecting the visual system

Until recently, the human visual system was thought to have an exquisitely contralateral representation, and was thought to be one of the least plastic parts of the brain (Tootell et al., 1998). However, there is now interest in visual function recovery after stroke (Brodtmann, 2012). Many therapists are taught that visual recovery after stroke is poor and incomplete, but results from natural history studies are at odds with this preconception. Whilst Ali et al. reported that less than 20% of completely hemianopic patients had full recovery of their visual fields after a month (Ali et al., 2013), more than 70% of those with partial hemianopia recovered their vision fully in the same time frame (Gray et al., 1989). Moreover, Zhang et al. found that spontaneous visual field defect recovery occurred in at least 50% of a group of 254 patients within 1 month of injury (Zhang et al., 2006a,b).

### Cross-modal plasticity in the visual system

Evidence for cross-modal plasticity in the visual system has emerged in the last decade, when functional neuroimaging researchers (using PET, TMS, and fMRI) revealed remarkable activation of the primary visual cortex during tactile tasks. Some regions in the visual system display high levels of cross-modality. There appear to be cross-modal interactions of auditory, visual, somatic, and even olfactory inputs, with studies in blind people demonstrating extraordinary levels of activation in occipital regions to auditory and tactile stimuli (Saenz et al., 2008; Collignon et al., 2009; Fiehler and Rosler, 2010; Lewis et al., 2010; Kupers et al., 2011; Wong et al., 2011; Striem-Amit et al., 2012). These findings represent some of the most exciting advances in cognitive neuroscience, as researchers explore the organizational principles that drive this cross-modal plasticity.

### Implications for therapy: restorative vs. compensatory training

Over the last few decades, a number of research groups postulated that deficits caused by acquired visual pathway injury in adulthood were potentially reversible through visual training strategies. Researchers demonstrated evidence of visual field recovery in people with stable lesions, often with lesions that were many years old (Kasten et al., 1999, 2006, 2007; Poggel et al., 2004, 2006, 2010; Henriksson et al., 2007; Chokron et al., 2008; Mueller et al., 2008; Jobke et al., 2009; Raemaekers et al., 2011). Visual Restoration Therapy (VRT) has been promoted by a number of researchers as a means of “striate system restoration” (Sabel and Kasten, 2000). The training usually consists of target detection during gaze fixation. During training, the subjects are asked to fix their gaze centrally whilst detecting stimuli in the border zone of their impaired visual field; i.e., on the edge of their visual field deficit. Results from these studies have demonstrated a gradual enlargement of their functional visual field, with concomitant reduction in their objective visual field deficit (Bergsma and van der Wildt, 2010).

It remains unclear whether such therapies are causing actual improvement of visual field deficits or whether they are adaptive, causing functional improvement by increased saccades into the affected hemifield (Reinhard et al., 2005; Glisson and Galetta, 2007). When the early studies were first published, many researchers felt that the functional improvements demonstrated by the authors were due to compensation strategies (e.g., better scanning and attention) rather than a true expansion of the visual field. However, field expansion has subsequently been shown to be independent of eye movements (Kasten et al., 2006). Mueller, Mast, and Sabel performed a large clinical observational study of 302 patients before and after being treated with computer-based VRT for a period of 6 months at eight clinical centers. In around 70% of patients, VRT was associated with a 17% improvement stimuli detection in the blind hemifield. These detection gains were not significantly correlated with eye movements, and were validated by standard perimetry (Mueller et al., 2007).

In their comprehensive review of spontaneous recovery and treatment effects following homonymous visual deficits, de Haan et al. identified important differences between the outcomes measured by restorative vs. compensatory training (de Haan et al., 2014). By definition, restorative training primarily focuses on improving the visual field, reducing the visual deficit, whilst compensatory training teaches the patient to apply scanning strategies in daily life in order to improve independence and mobility. They also recommended *patient participation measures* be used more frequently as a rehabilitation outcome, in order to assess whether an intervention or treatment is of benefit for their activities of daily living. These are important considerations for therapists working with patients with acquired visual field deficits, as merely expanding a visual field is of no benefit unless it translates into functional gains. Involvement of specialist therapists with specific expertise with clients with blindness or low vision is especially important in their rehabilitation.

## Concluding remarks

The data support the assumption that stroke patients have activation patterns comparable with healthy controls prior to their strokes, and that abnormal activation sites are a consequence of their pathology. The findings also demonstrate the degree of resilience and extent of plasticity in the visual system, with utilization of other visually sensitive areas even in the presence of sequential insults. Specialist therapies are available for treatment. Traditionally, these are largely compensatory training strategies, teaching the patient to overcome their deficit via compensatory methods. However, there is renewed interest in restorative training, although these methods are not without controversy. These data challenge the concept of a hard-wired visual system capable of minimal remodeling following injury. As demonstrated in this case report, mature primary visual cortex displays remarkable plasticity and capacity to reorganize following injury.

### Conflict of interest statement

The authors declare that the research was conducted in the absence of any commercial or financial relationships that could be construed as a potential conflict of interest.

## Supplementary material

Figure S1 **Schematic of activation task. (A)** Example of FACE image displaying white cross target, **(B)** 50% GRAY background with black central fixation cross, **(C)** Example of scrambled face (SCRF) image without white cross target, **(D)** 50% GRAY background displaying white cross target, **(E)** Example of FACE image without white cross target. First 100 s shown but cycle continued for 240 s.

## References

[B1] Ali M. Hazelton C. Lyden P. Pollock A. Brady M. (2013). Recovery from poststroke visual impairment: evidence from a clinical trials resource. Neurorehabil. Neural Repair 27, 133–141. 10.1177/1545968312454683 22961263

[B2] Astrup J. Siesjo B. K. Symon L. (1981). Thresholds in cerebral ischemia–the ischemic penumbra. Stroke 12, 723–725. 10.1161/01.STR.12.6.723 6272455

[B3] Bergsma D. P. van der Wildt G. (2010). Visual training of cerebral blindness patients gradually enlarges the visual field. Br. J. Ophthalmol. 94, 88–96. 10.1136/bjo.2008.154336 19692376

[B4] Bosley T. M. Dann R. Silver F. L. Alavi A. Kushner M. Chawluk J. B.. (1987). Recovery of vision after ischemic lesions: positron emission tomography. Ann. Neurol. 21, 444–450. 10.1002/ana.410210505 3496042

[B5] Brodtmann A. (2012). Vision, in Stroke Rehabilitation: Insights from Neuroscience and Imaging, ed Carey L. M. (Oxford: Oxford University Press), 173–185.

[B6] Brodtmann A. Puce A. Darby D. Donnan G. (2007). fMRI demonstrates diaschisis in the extrastriate visual cortex. Stroke 38, 2360–2363. 10.1161/STROKEAHA.106.480574 17600237

[B7] Brodtmann A. Puce A. Darby D. Donnan G. (2009a). Regional fMRI brain activation does correlate with global brain volume. Brain Res. 1259, 17–25. 10.1016/j.brainres.2008.12.044 19133239

[B8] Brodtmann A. Puce A. Darby D. Donnan G. (2009b). Serial functional imaging poststroke reveals visual cortex reorganization. Neurorehabil. Neural Repair 23, 150–159. 10.1177/1545968308321774 19029284

[B10] Brodtmann A. Puce A. Syngeniotis A. Darby D. Donnan G. (2003). The functional magnetic resonance imaging hemodynamic response to faces remains stable until the ninth decade. Neuroimage 20, 520–528. 10.1016/S1053-8119(03)00237-4 14527612

[B11] Calautti C. Baron J. C. (2003). Functional neuroimaging studies of motor recovery after stroke in adults: a review. Stroke 34, 1553–1566. 10.1161/01.STR.0000071761.36075.A6 12738893

[B12] Cao Y. Vikingstad B. S. George K. P. Johnson A. F. Welch K. M. A. (1999). Cortical language activation in stroke patients recovering from aphasia with functional MRI. Stroke 30, 2331–2340. 10.1161/01.STR.30.11.2331 10548667

[B13] Chokron S. Perez C. Obadia M. Gaudry I. Laloum L. Gout O. (2008). From blindsight to sight: cognitive rehabilitation of visual field defects. Restor. Neurol. Neurosci. 26, 305–320. 18997308

[B14] Collignon O. Voss P. Lassonde M. Lepore F. (2009). Cross-modal plasticity for the spatial processing of sounds in visually deprived subjects. Exp. Brain Res. 192, 343–358. 10.1007/s00221-008-1553-z 18762928

[B15] Darian-Smith C. Gilbert C. D. (1994). Axonal sprouting accompanies functional reorganization in adult cat striate cortex. Nature 368, 737–740. 10.1038/368737a0 8152484

[B16] de Haan G. A. Heutink J. Melis-Dankers B. J. Tucha O. Brouwer W. H. (2014). Spontaneous recovery and treatment effects in patients with homonymous visual field defects: a meta-analysis of existing literature in terms of the ICF framework. Surv. Ophthalmol. 59, 77–96. 10.1016/j.survophthal.2013.02.006 24112548

[B17] Dhamoon M. S. Moon Y. P. Paik M. C. Sacco R. L. Elkind M. S. (2012). Trajectory of functional decline before and after ischemic stroke: the Northern Manhattan study. Stroke 43, 2180–2184. 10.1161/STROKEAHA.112.658922 22649168 PMC3404224

[B18] Donnan G. A. Davis S. M. (2002). Neuroimaging, the ischaemic penumbra, and selection of patients for acute stroke therapy. Lancet Neurol. 1, 417–425. 10.1016/S1474-4422(02)00189-8 12849364

[B19] Duvernoy H. M. Bourgouin P. Cabanis E. A. Cattin F. (1999). The Human Brain: Surface, Three Dimensional Sectional Anatomy with MRI, and Blood Supply, 2nd Edn. New York, NY: Springer Verlag.

[B20] Fiehler K. Rosler F. (2010). Plasticity of multisensory dorsal stream functions: evidence from congenitally blind and sighted adults. Restor. Neurol. Neurosci. 28, 193–205. 10.3233/RNN-2010-0500 20404408

[B21] Glisson C. C. Galetta S. L. (2007). Visual rehabilitation: now you see it; now you don't. Neurology 68, 1881–1882. 10.1212/01.wnl.0000267412.54793.38 17536043

[B22] Goebel R. Muckli L. Zanella F. E. Singer W. Stoerig P. (2001). Sustained extrastriate cortical activation without visual awareness revealed by fMRI studies of hemianopic patients. Vision Res. 41, 1459–1474. 10.1016/S0042-6989(01)00069-4 11322986

[B23] Gray C. S. French J. M. Bates D. Cartlidge N. E. Venables G. S. James O. F. (1989). Recovery of visual fields in acute stroke: homonymous hemianopia associated with adverse prognosis. Age Ageing 18, 419–421. 10.1093/ageing/18.6.419 2629493

[B24] Henriksson L. Raninen A. Nasanen R. Hyvarinen L. Vanni S. (2007). Training-induced cortical representation of a hemianopic hemifield. J. Neurol. Neurosurg. Psychiatr. 78, 74–81. 10.1136/jnnp.2006.099374 16980334 PMC2117784

[B25] Jobke S. Kasten E. Sabel B. A. (2009). Vision restoration through extrastriate stimulation in patients with visual field defects: a double-blind and randomized experimental study. Neurorehabil. Neural Repair 23, 246–255. 10.1177/1545968308324221 19240199

[B26] Kasten E. Bunzenthal U. Muller-Oehring E. M. Mueller I. Sabel B. A. (2007). Vision restoration therapy does not benefit from costimulation: a pilot study. J. Clin. Exp. Neuropsychol. 29, 569–584. 10.1080/13803390600878919 17691030

[B27] Kasten E. Bunzenthal U. Sabel B. A. (2006). Visual field recovery after vision restoration therapy (VRT) is independent of eye movements: an eye tracker study. Behav. Brain Res. 175, 18–26. 10.1016/j.bbr.2006.07.024 16970999

[B28] Kasten E. Poggel D. A. Muller-Oehring E. Gothe J. Schulte T. Sabel B. A. (1999). Restoration of vision II: residual functions and training-induced visual field enlargement in brain-damaged patients. Restor. Neurol. Neurosci. 15, 273–287. 12671238

[B29] Khurshid S. Trupe L. A. Newhart M. Davis C. Molitoris J. J. Medina J.. (2012). Reperfusion of specific cortical areas is associated with improvement in distinct forms of hemispatial neglect. Cortex 48, 530–539. 10.1016/j.cortex.2011.01.003 21345430 PMC3125403

[B30] Kupers R. Beaulieu-Lefebvre M. Schneider F. C. Kassuba T. Paulson O. B. Siebner H. R.. (2011). Neural correlates of olfactory processing in congenital blindness. Neuropsychologia 49, 2037–2044. 10.1016/j.neuropsychologia.2011.03.033 21458471

[B31] Lewis L. B. Saenz M. Fine I. (2010). Mechanisms of cross-modal plasticity in early-blind subjects. J. Neurophysiol. 104, 2995–3008. 10.1152/jn.00983.2009 20668272 PMC3007643

[B32] Mueller I. Gall C. Kasten E. Sabel B. A. (2008). Long-term learning of visual functions in patients after brain damage. Behav. Brain Res. 191, 32–42. 10.1016/j.bbr.2008.03.005 18436312

[B33] Mueller I. Mast H. Sabel B. A. (2007). Recovery of visual field defects: a large clinical observational study using vision restoration therapy. Restor. Neurol. Neurosci. 25, 563–572. 18334773

[B34] Nelles G. Widman G. de Greiff A. Meistrowitz A. Dimitrova A. Weber J.. (2002). Brain representation of hemifield stimulation in poststroke visual field defects. Stroke 33, 1286–1293. 10.1161/01.STR.0000013685.76973.67 11988605

[B35] Poggel D. A. Kasten E. Muller-Oehring E. M. Bunzenthal U. Sabel B. A. (2006). Improving residual vision by attentional cueing in patients with brain lesions. Brain Res. 1097, 142–148. 10.1016/j.brainres.2006.04.011 16777076

[B36] Poggel D. A. Kasten E. Sabel B. A. (2004). Attentional cueing improves vision restoration therapy in patients with visual field defects. Neurology 63, 2069–2076. 10.1212/01.WNL.0000145773.26378.E5 15596752

[B37] Poggel D. A. Mueller I. Kasten E. Bunzenthal U. Sabel B. A. (2010). Subjective and objective outcome measures of computer-based vision restoration training. Neurorehabilitation 27, 173–187. 10.3233/NRE-2010-0594 20871147

[B38] Raemaekers M. Bergsma D. P. van Wezel R. J. van der Wildt G. J. van den Berg A. V. (2011). Effects of vision restoration training on early visual cortex in patients with cerebral blindness investigated with functional magnetic resonance imaging. J. Neurophysiol. 105, 872–882. 10.1152/jn.00308.2010 21160012

[B39] Reinhard J. Schreiber A. Schiefer U. Kasten E. Sabel B. A. Kenkel S.. (2005). Does visual restitution training change absolute homonymous visual field defects? A fundus controlled study. Br. J. Ophthalmol. 89, 30–35. 10.1136/bjo.2003.040543 15615742 PMC1772456

[B40] Sabel B. A. Kasten E. (2000). Restoration of vision by training of residual functions. Curr. Opin. Ophthalmol. 11, 430–436. 10.1097/00055735-200012000-00008 11141637

[B41] Saenz M. Lewis L. B. Huth A. G. Fine I. Koch C. (2008). Visual motion area MT+/V5 responds to auditory motion in human sight-recovery subjects. J. Neurosci. 28, 5141–5148. 10.1523/JNEUROSCI.0803-08.2008 18480270 PMC3165167

[B42] Schoenfeld M. A. Noesselt T. Poggel D. Tempelmann C. Hopf J. M. Woldorff M. G.. (2002). Analysis of pathways mediating preserved vision after striate cortex lesions. Ann. Neurol. 52, 814–824. 10.1002/ana.10394 12447936

[B43] Shirani P. Thorn J. Davis C. Heidler-Gary J. Newhart M. Gottesman R. F.. (2009). Severity of hypoperfusion in distinct brain regions predicts severity of hemispatial neglect in different reference frames. Stroke 40, 3563–3566. 10.1161/STROKEAHA.109.561969 19762699 PMC2790042

[B44] Smith S. M. (2002). Fast robust automated brain extraction. Hum. Brain Mapp. 17, 143–155. 10.1002/hbm.10062 12391568 PMC6871816

[B45] Striem-Amit E. Dakwar O. Reich L. Amedi A. (2012). The large-scale organization of “Visual” streams emerges without visual experience. Cereb. Cortex 22, 1698–1709. 10.1093/cercor/bhr253 21940707

[B46] Thulborn K. R. Carpenter P. A. Just M. A. (1999). Plasticity of language-related brain function during recovery from stroke. Stroke 30, 749–754. 10.1161/01.STR.30.4.749 10187873

[B47] Tootell R. B. Mendola J. D. Hadjikhani N. K. Liu A. K. Dale A. M. (1998). The representation of the ipsilateral visual field in human cerebral cortex. Proc. Natl. Acad. Sci. U.S.A. 95, 818–824. 10.1073/pnas.95.3.818 9448246 PMC33803

[B48] Wong M. Gnanakumaran V. Goldreich D. (2011). Tactile spatial acuity enhancement in blindness: evidence for experience-dependent mechanisms. J. Neurosci. 31, 7028–7037. 10.1523/JNEUROSCI.6461-10.2011 21562264 PMC6703211

[B49] Zhang X. Kedar S. Lynn M. J. Newman N. J. Biousse V. (2006a). Homonymous hemianopias: clinical-anatomic correlations in 904 cases. Neurology 66, 906–910. 10.1212/01.wnl.0000203913.12088.93 16567710

[B50] Zhang X. Kedar S. Lynn M. J. Newman N. J. Biousse V. (2006b). Natural history of homonymous hemianopia. Neurology 66, 901–905. 10.1212/01.wnl.0000203338.54323.22 16567709

