# Supplementary figures and images for: Extrastriate visual cortex reorganizes despite sequential bilateral occipital stroke: implications for vision recovery
Source: Front Hum Neurosci. 2015 Apr 28;9:224. doi: 10.3389/fnhum.2015.00224 (PMC4412053; doi:10.3389/fnhum.2015.00224)

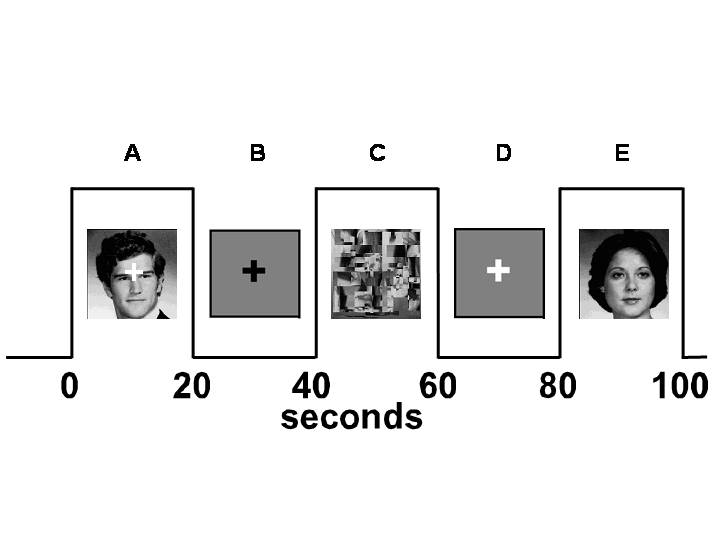

Supplement: Figure S1 — Schematic of activation task. (A) Example of FACE image displaying white cross target, (B) 50% GRAY background with black central fixation cross, (C) Example of scrambled face (SCRF) image without white cross target, (D) 50% GRAY background displaying white cross target, (E) Example of FACE image without white cross target. First 100 s shown but cycle continued for 240 s. [file Image1.JPEG]
